# Book Notices

**Published:** 1866-01

**Authors:** 


### BOOK NOTICES.

*The Practice of Medicine and Surgery Applied to the Diseases and Accidents Incident to Women.* By W. H. BYFORD, A. M., M. D., author of "A Treatise on the Chronic Inflammations and Displacements of the Unimpregnated Uterus," and Prof. of Obstetrics and Diseases of Women and Children in the Chicago Medical College. Philadelphia: Lindsay & Blackiston. 1865.

The very favorable manner in which this work has been received by the medical press, must be regarded as highly flattering to the author. Prof. Byford has long been known as a teacher, and as a practitioner of much experience in the particular branch to which he has devoted the best energies of his life. This work has extended the reputation of its author far beyond the immediate sphere of his labors.

Woman, not only from her peculiar organization, but also from the habits, which society imposes upon her almost in spite of herself, is too often the victim of severe and almost lifelong suffering. To mitigate this suffering as far as possible, should be the earnest aim of every physician. This volume, we believe, is the latest work on diseases of women published, either in Europe or America. Its high order of merit commends it to the special notice of all practitioners.

---

*Rhynoscopy and Laryngoscopy.* By Dr. FRIEDERICH SEMELEDER, Physician in Ordinary to His Majesty the Emperor of Mexico, Member of the Royal Medical Society of Vienna, and of the Medical Society of the Pantheon in Paris, etc., etc. Translated from the German by EDWARD T. CASWELL, M. D. New York: William Wood & Co, 61 Walker st.

This is an elegant little volume which reflects credit upon author, translator and publisher. The two monographs which it contains present a valuable summary of the knowledge which has been obtained by the most patient observation with the rhynoscopic and laryngoscopic mirror. The difficulties which oppose investigation by these methods are fully set forth, and the positive results which are attainable are not presented in such a way as to dazzle and to mislead the student. The author does not hesitate to confess that laryngoscopic pathology and surgery are as yet very incomplete; and in many instances, with the tranquility of a true philosopher, he awaits the further multiplication of observations before announcing any conclusion, or committing himself to any hasty generalization.

The examination by the aid of reflected light of cavities into which, before the days of Czermak, the eye could not penetrate, marks a long stop in advance towards that degree of accuracy in diagnosis which is the aim of every scientific observer. Nor can it be urged that the laryngoscope, like the opthalmoscope, too often assists only in diagnosis without aiding therapeutical effort. The cases recorded on the pages of this work afford a sufficient refutation of such an objection. Aside from the chance for pathological study which are thus discovered, it is no small thing to be able to investigate the condition of living mucous tissue in parts so far removed from ordinary observation. The demonstrations of the dissecting room cannot compare with the exhibitions which are thus made of organs and tissues through which the current of life is flowing. On physiological grounds alone, then, the mirror of Czermak every day introduces us to fields of study as interesting as that once afforded by the fistula of Alexis St. Martin.

We copy the following history from Dr. Semeleder's pages:

A lady (her age I did not ask), a governess, came to me last autumn to be examined and to ask my advice. She had suffered for five years from complete aphonia, which had been gradually developed. On a careful examination, I discovered three formations of various sizes. The largest of these was spherical and was seated in the vicinity of the left vocal chord; a second, smaller and club-shaped, projected from the anterior angle of the glottis, and lay with its free extremity upon the first; the third and smallest protruded from the anterior surface of the right arytenoid cartilage, at about the level of the vocal chord, and extended into the latter structure.*

I stated to her that I was inclined to operate; that I could not insure success, so far as the restoration of the voice was concerned; but that by the operation, even as regarded the voice, nothing was to be lost, since she was already voiceless, a

* For convenience of reference these will be designated respectively as Nos. 1, 2 and 3. (Tr.)

circumstance sufficiently unfortunate for a governess. To my amazement, I must confess, there was no dyspnœa, not even an abnormal murmur to be heard on auscultation.

The formation had at first, from its pale, reddish-yellow color, from its dim lustre, and from its uneven raspberry-like surface, led me to regard it as an enchondromatous or a fibroid tumor, but as I studied it more carefully with reference to a future operation, I found by the aid of the sound that it had something of the consistency of flesh, and, finally, I concluded that it must be composed of areolar tissue. The largest of the tumors proceeded, as I have said, from the left side of the ventricle of the larynx, and covered the left vocal chord in such a manner that only a small portion of the chord could be seen; this fragment seemed to have a normal appearance. The anterior extremity of the left vocal chord was covered by the polyp No. 2. As the patient was so sensitive under the examination, I could not determine whether the polyp No. 1 lay free upon the left vocal chord, or whether it was intimately connected with it. When the glottis was closed all three of the polypi were shoved over each other. Thus much, however, I could observe: By the attempt to utter sounds, the polyp No. 1 was rolled up around its broad basis, so that it would then lie upon the right vocal chord, and would be wedged in between the ventricular and the vocal chords of both sides. The polypi Nos. 2 and 3 proved to be quite freely moveable, following the respiratory current; No. 2 particularly, with its free end glided down over No. 1, and was again thrown upwards by a forcible expiration.

The patient, aside from this local trouble, was apparently in perfect health, and there were no reasonable grounds for supposing a connexion between the local disease and any cachexia, or the pre-existence of any special disturbing cause.

On the first of November, 1863, I undertook the operation, after having forewarned the lady that she must have a large stock of patience, and must expect to undergo a second operation. The operation itself was undertaken after the same preliminaries as I have described in connexion with the first case, viz. the local applications of morphia and chloroform, the fixation of the patient's head by a trustworthy assistant, and of the tongue by the patient's own fingers, Wigtrich's globe apparatus on a petroleum lamp, the operating spectacles above mentioned, and the laryngeal mirror held loosely in the left hand. The efforts to produce anæsthesia were made at very short intervals for the space of two hours, but with very imperfect success; the epiglottis forceps could not be tolerated.

I removed, by means of the polyp forceps, properly adjusted, the formations Nos. 2 and 3, *i. e.* the one seated at the anterior angle of the glottis, and the one upon the right arytenoid cartilage, leaving of the former only a very small stump; accomplishing it, to be sure, only after frequent attempts. The greater part of the polyp No. 2 was removed by a single fortunate seizure; the wedge-shaped growth was about 1½ centimetres in length, and was first separated from its attachment, as the forceps which had seized it had reached the edge of the epiglottis.

Only a small portion of the polyp No. 3 was drawn out; the rest fell down into the trachea and was lost. The bleeding and the pain were very slight; and there was scarcely any reaction. The polyp seemed to be quite hard. The patient was very much amazed that there was still no improvement in her voice, but I, for my part, could not be much surprised.

The microscopic examination of the portions removed, made by Dr. Schott, showed again newly developed areolar tissue, with large loops of vessels and epithelium.

On the 15th of November, 1863, I applied myself to the removal of the largest polyp No. 1, with the same preparations as before. I now knew that its consistency was quite compact, and was therefore convinced that the operation this time would prove very difficult; for it seemed probable beforehand that I should not be able to seize the polyp with the forceps on account of its remarkable size and rounded form. I determined, however, to make the attempt, and, if my suspicions proved correct, to divide the tumor with a knife, and finally remove it in fragments with the forceps. This soon became necessary. While the tumor, by a continued effort on the part of the patient to utter sound, was held firmly, I succeeded, after one or two attempts, in making, with the aid of the mirror, two cuts, using Leiter's knife, after having inserted a lancet blade. The edges of the cut bled but little, and they did not gape at all.

I then applied the forceps, and removed, piecemeal, the greater part of this polyp. On the larger of the removed pieces, the smooth surface of the cut could be distinctly seen. The bleeding was slight; altogether a couple of teaspoonfuls might have been raised, little by little, mingled with mucus. The operation proved very wearisome for all of us, having lasted, inclusive of the attempts to produce anæsthesia, almost four hours. Still the reaction was limited to a slight pain in the larynx, which lasted about three days. The microscopic examination showed the same results as before.

When I next examined the patient, of the large polyp there was only a remnant of about the breadth of the vocal chord, with a projecting lobe on the posterior portion. On the anterior angle of the glottis, a small nodule had been reproduced. To the astonishment of both myself and the patient, the voice unfortunately was not in the slightest degree improved. I now began to fear that the stump of the large polyp might have been so attached to the left vocal chord, or grown out of it in such a manner, to the removal of the remnant without injury to the vocal chord would be quite impossible. So I contented myself with blowing into the larynx most assiduously pulverized alum, hoping to produce shrinking of the remnant of the polyp. But it was all in vain; the voice was not restored. In laughing as well as in quick inspiration, a short dull tone could be elicited, but that was all. Occasionally in speaking the sound was somewhat rougher, but the change was only very transient. I now sought to destroy the stump of the polyp by cauterization, and twice applied nitrate of silver in substance; eschars were formed on the spots touched, but the voice did not come back. For me it was a painful moment; the patient must be encouraged to hope on, and I, for one, had nearly given up all hope. In the meantime, on the 31st of December, 1863, and on the 2d of January, 1864, I removed from the anterior angle of the glottis the lobe on the posterior extremity of the stump of the large polyp, and also the small growth which had recently sprouted. Both of these operations were conducted without assistance, and without any preliminary preparation. The lobe was as large as a small pea. I could not say why the voice did not yet return.

Meanwhile it occurred to me that when the patient had been left to herself for a couple of days, the interior of the larynx, especially the right vocal chord, was found somewhat pallid, and also that the sound of the voice was somewhat better. Hence I supposed that the larynx, in consequence of the perpetual blowing of powder, the cauterizations, and the instrumental applications, must have been kept in a continual state of irritation, which of itself would at last affect injuriously the formation of the voice. I determined, therefore, to try non-interference with the larynx for a period of eight days, with the firm resolution at the end of that time, if no improvement had taken place, to resort to a combined operation with the knife and forceps. It was evident that this operation would now be much more difficnlt, as the stump was very narrow and flat, and hence that the line of my incision must be kept close to that of the ventricular chord, while at the same time I must avoid injuring it; and yet if I should succeed in all this, and still find the polyp firmly attached to the vocal chord, all my labor would be in vain, and the voice would be lost forever. Although as an operator I had every ground to be satisfied with my experience thus far, yet, in the opinion of my patient and of the public, both I and my art would be objects of reproach if the voice was not restored. The last trump was, therefore, to be carefully played.

I therefore recommended my patient, who coincided with all my suggestions, to visit me again after the lapse of a few days, when we would come to a final conclusion. Now, I might as well confess that I was most agreeably surprised at the next visit, after an interval of several days, to hear my patient address me in a voice somewhat hollow and not metallic, but yet quite good and clear. The lady told me that for a year and a half she had not spoken so well as within the last two or three days, but that she was easily tired, and then her voice again became hoarse. An examination showed me that in consequence of the numerous and repeated applications, a superficial ulcer had been developed on the surface of the stump, for which I hope for a still further diminution of the remnant of the polyp. If the voice should not improve, and the patient should not be satisfied with its present condition, I shall be inclined to operate once more.

Dr. Caswell deserves the thanks of the profession for the neatness of his translation. It is seldom that he has permitted the intrusion of a phrase or even of a word to remind one that this is not an original English work. It is only the well ordered movement of the author's ideas that points to an art beyond that of the majority of our own medical writers.

---

*Lectures on the Diseases of Infancy and Childhood.* By CHARLES WEST, M. D., Fellow of the Royal College of Physicians; Physician to the Hospital for Sick Children. Fourth American from the Fifth Revised and Enlarged English Edition. Philadelphia: Henry C. Lea. 1866.

The great reputation of Dr. West in this country and Europe as a teacher, and as an elegant writer—the fact that his work on diseases of children has passed through so many editions in English and German, and that translations have been demanded in Dutch, French, Danish and Prussian, renders superfluous any extended notice of this, the fourth American edition. Every practising physician should make this work a careful study. We would especially commend the lecture on Diphtheria as a concise and yet faithful statement of the known facts regarding this dangerous disease. For sale by W. B. Keen & Co., Lake st.

*Sixth and Seventh Annual Report of the Chicago Charitable Eye and Ear Infirmary,* 16 *East Pearson Street, presented by the Board of Surgeons, for the year ending May* 1, 1864, *and for the year ending May* 1, 1865.

In accordance with the Constitution and By-Laws of the Association, the Surgeons would most respectfully report:

That during the year ending May 1st, 1864, four hundred and forty-four, and during the year ending May 1st, 1865, four hundred and fifty-eight patients have been under treatment,—making an aggregate of two thousand one hundred and twenty-six that have been treated since the opening of the Infirmary in 1858.

The following is a classified list of the diseases which have been treated during the past two years:

DISEASES OF THE EYE.

| | | | |
|---|---|---|---|
| Wounds and Injuries | 32 | Diseases of the Retina and Optic Nerve | 29 |
| Conjunctivitis, catarrhal | 57 | Diseases of the Choroid and Sclerotic | 14 |
| " granular | 123 | Opacity of the Vitreous Humor | 7 |
| " " with vascular cornea | 120 | Glaucoma | 1 |
| Conjunctivitis neonatorum | 8 | Diseases of the Lens — Cataract and Injuries | 31 |
| " purulent | 19 | Diseases of the Muscles | 13 |
| " pustular | 64 | Abnormal Accommodation | 5 |
| Diseases of the Cornea | 121 | Unclassified | 23 |
| Foreign bodies on Cornea | 11 | | |
| Diseases of the Lids | 96 | Total | 813 |
| " " Iris | 25 | | |
| " " Lachrymal Apparatus | 16 | | |

DISEASES OF THE EAR.

| | | | |
|---|---|---|---|
| Impacted Cerumen | 9 | Perforation of Membrani Tympani | 4 |
| Otorrhœa | 35 | Polypus | 1 |
| Inflammation of External Meatus | 8 | Unclassified | 21 |
| Inflammation of Membrana Tympani | 11 | Total | 89 |

It will thus be seen that a larger number of patients have sought advice and treatment at the Infirmary during the past than any previous year. As a proof of the continued confidence with which its efforts are elsewhere regarded, it may be stated, that the expenses of patients from a distance have, not unfrequently, been borne by benevolent individuals in their respective communities. Physicians, also, in various parts of the Northwest have expressed warm interest in the welfare of this charity, and have frequently testified to the great good it has accomplished.

The benefit which many of these patients have received is incalculable. Parents, unfortunate and helpless, have been restored to sight and the means of supporting their families. A large number of children have been rescued, it is believed, from partial or total blindness—and thus from life-long poverty. Many patients, affected with diseases, trifling in themselves, but which, if neglected, are almost certain to result in permanent injury of vision, have been relieved by short and simple treatment. And it is upon this class of patients, that the surgeons look with peculiar interest, as illustrating the manner in which this charity, if possible, accomplishes most good, by encouraging poor patients, without fear of expense, to *seek medical aid in the very commencement of their diseases, before they have assumed a dangerous form.*

There is scarcely any form of charity, whose claims can be so forcibly urged on the grounds of humanity and economy, as this. It relieves physical suffering and mental distress by the cure of painful diseases, and by removing fears of threatened blindness; it restores many with impaired vision to sight, and to their daily labors, thereby removing one cause of poverty; it prevents ignorance by rescuing poor children from partial or total loss of sight—thus enabling them to acquire the rudiments of knowledge, and to follow in after life honorable and remunerative occupations. How much more in accordance with an enlightened humanity is it to relieve such children, than to neglect them till they become dependent upon the Blind Asylum for their education and limited means of support.

On the ground of economy this charity claims *especial* consideration. So far as it prevents blindness—so far it lessens taxation by reducing the number of the poor dependent upon public or private charity—and so far it adds to the productive labor and wealth of the State. Its principle is Prevention, one of the first laws of Economy. It does not encourage the habit of idleness and begging, as, it is feared, is too often the case with direct pecuniary aid. It restores to health, hope and useful activity. It would be difficult to point to another form of charity by which so much good can be accomplished at so little cost.

The Surgeons are most happy to inform the Association and the public, that W. L. Newberry, Esq., President of the Board of Trustees, has generously donated the lease of a valuable lot of land for the term of ten years. A commodious building has already been placed upon this lot, and furnished for the use of the Infirmary.

The Surgeons take pleasure also in stating that the Infirmary was incorporated in February, 1865, by a special act of the General Assembly of the State of Illinois.

This charity is intended for the poor of the Northwest, as well as of Chicago. For such patients, treatment, medicines and lodging will be provided gratuitously. A charge is necessary at present for board, since the means of the Infirmary are insufficient to furnish this last without charge. Efforts will be made to establish a fund by which the poor may also obtain gratuitous board.

In entering upon this new era of its history, may not the Infirmary receive such aid and encouragement as its extending usefulness and increasing needs require.

TRUSTEES.

WALTER L. NEWBERRY, *President.*
LUTHER HAVEN, *Vice-President.*
S. STONE, *Secretary.*
E. B. McCAGG, *Treasurer.*
W. H. BROWN,
WILLIAM BARRY,
FLAVEL MOSELEY,
WESLEY MUNGER,
E. W. BLATCHFORD,
CYRUS BENTLEY,
T. B. BRYAN,
P. CARPENTER.

SURGEONS.

*Attending.*
EDWARD L. HOLMES, M. D.,
EDWIN POWELL, M. D.

*Consulting.*
PROF. D. BRAINARD, M. D.,
PROF. J. W. FREER, M. D.

MR. J. G. RICHARDS, *Superintendent.*
MRS. RICHARDS, *Matron.*

---

We are requested to announce that the Surgical Clinics of Rush Medical College Dispensary will be conducted by Dr. Edwin Powell after the close of the lectures, and through the coming spring and summer. Cases from the country will be attended to on Saturdays.

---

DIED.—At his residence in Leslie, Michigan, after a protracted illness of typhoid fever, H. F. EATON, a student of Rush Medical College, in the twenty-fifth year of his age.

